# Spontaneous dimerization of the hepatitis C virus 3′X RNA

**DOI:** 10.1016/j.jbc.2025.110963

**Published:** 2025-11-20

**Authors:** Parker D. Sperstad, Erik D. Holmstrom

**Affiliations:** 1 Department of Molecular Biosciences, University of Kansas, Lawrence, Kansas, USA; 2 Department of Chemistry, University of Kansas, Lawrence, Kansas, USA

**Keywords:** Hepatitis C virus, noncoding RNA, single-molecule FRET, dimerization, kinetics

## Abstract

The 3ʹX RNA of the hepatitis C virus is a highly conserved, 98-nucleotide, non-coding sequence located at the 3ʹ terminus of the viral genome. This essential riboregulatory element contains a 16-nucleotide sequence, known as the dimer linkage sequence (DLS), which has been shown *in vitro* to facilitate the dimerization of the first 55 nucleotides of 3ʹX (3ʹX55). Here, we employ a novel integrative approach based on quantitative single-molecule FRET and analytical size-exclusion HPLC to monitor the structural, energetic, and dynamic aspects of 3ʹX55 dimerization. At high RNA concentrations, we see that 3′X55 can adopt multiple dimeric species with different structural properties. The interconversion between these species is slow, requiring several hours to reach equilibrium. Finally, the concentration- and time dependence of dimerization are both well described by a simple four-state kinetic model, which may explain how this riboregulatory interaction governs critical RNA-dependent processes during viral replication.

In 2021, the World Health Organization estimated that there were nearly 60 million cases of the liver inflammatory disease hepatitis C. This disease is caused by the hepatitis C virus (HCV), with chronic infections often leading to cirrhosis and liver cancer. This bloodborne pathogen is a ∼ 50 nm lipid-enveloped particle decorated with viral glycoproteins (1, 2, 3, 4, 5, 6) (Fig. 1*A*). The core of this particle consists of numerous copies of the viral nucleocapsid protein condensed around the ∼ 9600-nucleotide positive-sense single-stranded RNA genome (7, 8, 9). While the biological functions of the first two parts of the genome—the 5′ untranslated region (UTR) (10, 11) and the open-reading frame (8, 12, 13)—have both been well-established, the importance of the terminal 3′UTR is yet to be fully resolved. Investigations of this untranslated region have primarily focused on a highly conserved 98-nucleotide sequence at the tail end of the genome referred to as the 3′X RNA (14, 15, 16, 17, 18, 19) (Fig. 1*B*).

**Figure 1:**
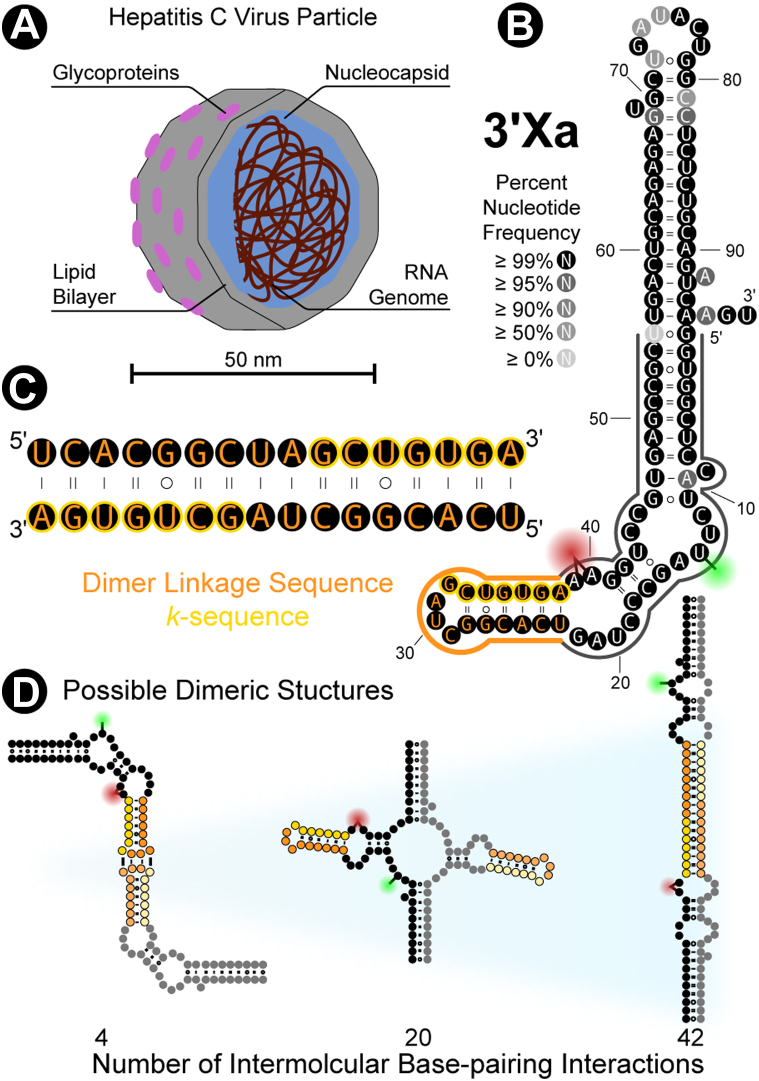
**The 3′X RNA of the Hepatitis C Virus.***A*, schematic of the hepatitis C virus highlighting the structural components of the particle. *B*, the highly conserved 3′X RNA can adopt a two stem-loop conformation (3′Xa) that sequesters the *k*-sequence (*yellow*) while also orienting the Dimer Linkage Sequence (DLS; *orange*) as an apical stem-loop. The structural, energetic, and kinetic aspects of dimerization were characterized by studying the first 55 nucleotides of 3′X (3′X55; *outlined*) with size-exclusion HPLC and single-molecule FRET. The donor fluorophore (Cy3B; *green*) was attached to nucleotide 13, and the acceptor fluorophore (CF660R; *red*) was attached between nucleotides 39 and 40. *C*, the *k*-sequence is nested within the 16 nucleotides that make up the self-complementary DLS. *D*, three examples of possible secondary structures of dimeric 3′X55 with increasing numbers of intermolecular base-pairing interactions (*left* to *right*).

As a monomer, 3′X can adopt two conformations, wherein the first 55 nucleotides adopt entirely distinct secondary structures (3′Xa and 3′Xb) (18, 20, 21, 22, 23). The 3′Xa conformation sequesters most of the 7-nucleotide *k*-sequence (Fig. 1, *B* and *C*; *yellow*) *via* intramolecular base-pairing interactions (18, 24). This obstruction limits these nucleotides from participating in an RNA–RNA interaction network implicated in the regulation of viral protein and RNA synthesis (25, 26, 27). Interestingly, the *k*-sequence is nested within the 16-nucleotide Dimer Linkage Sequence (DLS) of 3′X (Fig. 1, *B* and *C*; *orange*). The self-complementary nature of the DLS allows these sequences to dimerize *via* the formation of various intermolecular base-pairing interactions (Fig. 1*D*) with the same sequences of another copy of the genome (22, 23, 28, 29). Recently, dimerization has been hypothesized to disrupt the riboregulatory network associated with the *k*-sequence, thereby shutting down both viral protein and RNA synthesis, and ultimately allowing for the assembly of infectious viral particles (18, 30). Nevertheless, the product(s), mechanism(s), and biological function(s) of dimerization have yet to be fully resolved.

Here, we use quantitative single-molecule Förster Resonance Energy Transfer (FRET) spectroscopy in conjunction with analytical size-exclusion High-Performance Liquid Chromatography (HPLC) to investigate the structural, energetic, and dynamic properties of dimerization, focusing specifically on the first 55 nucleotides of 3′X (3′X55). FRET is a highly distance-dependent photophysical phenomenon routinely exploited to spectroscopically monitor the molecular dimensions of fluorescently labeled biomolecules under a wide range of solution conditions (31, 32, 33). When conducted at the single-molecule level, FRET-based experiments can characterize individual members of a heterogeneous population, which can be challenging to accomplish for many ensemble experiments. Furthermore, single-molecule FRET utilizes extremely low concentrations (∼100 pM) of fluorescently labeled biomolecules, which, in this work, facilitates observation of monomeric 3′X55 without having to introduce mutations that disrupt dimerization (18, 23, 28). We also use size-exclusion HPLC to monitor dimerization *via* changes in the hydrodynamic volumes (34) of various 3′X55 conformers (35). Conveniently, these two approaches probe orthogonal structural properties (*i.e.*, distances and volumes), which, when used together, help offset any shortcomings they may have when used in isolation.

Our findings demonstrate that 3′X55 dimerization occurs spontaneously at room temperature, yielding both compact dimeric conformations (CD) and extended dimeric conformations (ED) that were characterized using these two complementary approaches. Interestingly, MgCl_2_ is required for the formation of CD; however, it is not essential for the formation of ED. The extent of dimerization is greatly dependent on the concentration of 3′X55, and the rate of this process is slow, taking several hours to reach equilibrium. Finally, a simple four-state kinetic model for dimerization explains how this conformational change may regulate several critical RNA-dependent processes during viral replication.

## Results

The HCV 3′X RNA has previously been shown to dimerize with itself and is expected to form intermolecular complexes (Fig. 1*D*) involving the first 55 nucleotides of each monomer (22, 28, 29). However, the mechanism of dimerization and the conformations of the resulting species have not yet been thoroughly resolved. Here, we characterize the structural, energetic, and dynamic properties of dimerization using single-molecule FRET and size-exclusion HPLC, the former of which requires fluorescently labeled RNA. The labeled RNA constructs (^L^3′X55) described in this work were used previously to uncover the slow conformational dynamics of monomeric 3′X55 (18). The donor (Cy3B) and acceptor (CF660R) fluorophores are coupled to primary amines located on U13 and after A39 (Fig. 1*B*), both of which are in unpaired regions of the RNA (18).

### Refolded 3′X55 is monomeric

**Figure 2:**
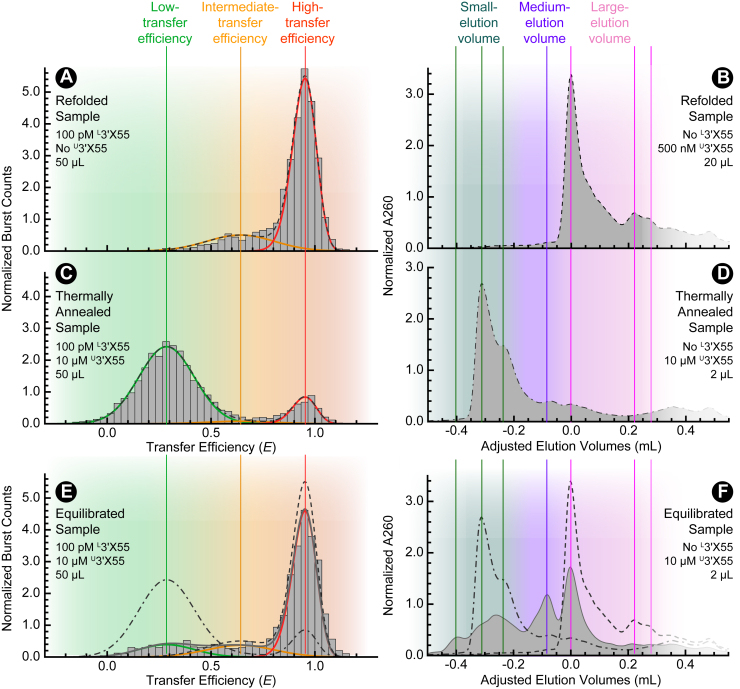
**Single-molecule FRET and Size-exclusion HPLC of 3′X55.***A*, when refolded, labeled 3′X55 (^L^3′X55) adopts two populations, a minor one at intermediate-transfer efficiency values (*orange*) and a major one at high-transfer efficiency values (*red*). *B*, when refolded, unlabeled 3′X55 (^U^3′X55) elutes at large-elution volumes (*pink*) with minor trailing chromatographic features. *C*, when thermally annealed in the presence of ^U^3′X55, ^L^3′X55 adopts a low-transfer efficiency (*light green*) population. *D*, when thermally annealed, ^U^3′X55 elutes at small-elution volumes (*dark green*) well before *aEV* = 0 ml. *E*, when equilibrated under baseline experimental conditions in the presence of ^U^3′X55, ^L^3′X55 samples all three FRET populations observed in (*A* and *C*). *F*, when equilibrated under baseline experimental conditions, ^U^3′X55 elutes at many different volumes, including those associated with (*B* and *D*), as well as at a medium-elution volume (*purple*). The refolded and thermally annealed reference samples are shown as *broken lines*.

A rigorous investigation of dimerization requires foundational knowledge of the isolated monomers of 3′X55 (18). Here, we both validated and expanded upon these insights. First, ^L^3′X55 was refolded at a dilute concentration of 10 nM (see experimental procedures) to capture the molecules in their monomeric forms. Next, fluorescence time trajectories were recorded for samples containing single-molecule concentrations (*e.g.*, 100 pM) of refolded ^L^3′X55 under baseline experimental conditions (*i.e.*, 1 mM MgCl_2_, 150 mM NaCl, 25 mM HEPES, 12.5 mM NaOH). When the fluorophores pass through the focus of our excitation sources, they emit a ∼ 1 ms burst of photons that transiently exceeds background levels. The photons from these events are then used to calculate transfer efficiencies for thousands of bursts over the course of 10 min, which are ultimately compiled into a histogram to quantify the resulting distributions of transfer efficiencies. Consistent with our previous results (18), the transfer efficiency histograms of these refolded samples indicate that ^L^3′X55 predominantly adopts two monomeric conformations (Fig. 2*A*): an intermediate-transfer efficiency conformation (3′X55b) located at ⟨*E*⟩ ≈ 0.64 and a high-transfer efficiency conformation (3′X55a) located at ⟨*E*⟩ ≈ 0.96. To characterize the global conformations of the monomeric ^L^3′X55 in the refolded (*RF*) samples, we used fluorescence correlation spectroscopy (FCS) analyses to determine that the average diffusion time under baseline experimental conditions was $\left\langle \tau_{D}\right\rangle$_*RF*_ ≈ 0.35 ± 0.01 ms. Additionally, we used polyacrylamide gel electrophoresis and size-exclusion HPLC to assess the electrophoretic mobility and hydrodynamic volume of unlabeled 3′X55 (^U^3′X55) in the refolded samples. For gel electrophoresis experiments under baseline experimental conditions, the refolded RNA appears to travel as monomeric bands (Fig. S1*A*; *lane* 1 *and 2*). In size-exclusion HPLC, the refolded RNA elutes as a dominant peak at a large elution volume of ∼ 2.6 ml, with two chromatographic features apparent in the next ∼ 0.3 ml of eluent (Fig. 2*B*). Because we intended to use the chromatographic features of the monomeric RNA in these refolded samples as a point of reference for dimerization, the elution volume values of all chromatograms were adjusted (see experimental procedures) such that this peak was positioned at an adjusted elution volume of *aEV* = 0 ml (Fig. 2*B*).

Combined, the above single-molecule FRET, gel electrophoresis, and size-exclusion HPLC results (Fig. 2, *A* and *B*) robustly characterize the monomeric forms of 3′X55 that exist at low RNA concentrations immediately after refolding. Any observations at high concentrations of ^U^3′X55 that deviate from this behavior are likely the result of intermolecular interactions (*e.g.*, dimerization).

### Thermal annealing produces extended dimeric conformations

To initially assess the ability of the 3′X55 to form dimeric structures, we first thermally annealed (see experimental procedures) ^L^3′X55 (100 pM) in the presence of unlabeled ^U^3′X55 (10 μM) to form single-molecule concentrations of FRET-labeled heterodimers. Transfer efficiency histograms from these thermally annealed (*TA*) samples under baseline experimental conditions (Fig. 2*C*) depict a new, low-transfer efficiency population located at ⟨*E*⟩ ≈ 0.29, which we attribute to the formation of extended dimeric conformations (ED) of 3′X55 that are comprised of many intermolecular base-pairing interactions (Fig. 1*D*). To further assess the oligomeric status of ^L^3′X55, we again used FCS analyses to determine that the average diffusion time was $\left\langle \tau_{D}\right\rangle$_*TA*_ ≈ 0.48 ± 0.01 ms for thermally annealed samples. As expected, this value was larger than that of the refolded samples by a factor of ∼ ∛2, which roughly corresponds to a doubling in mass according to the Stokes-Einstein relationship.

When monitored *via* gel electrophoresis, a sizable portion of the RNA in the thermally annealed samples now travels as a dimeric band (Fig. S1*A*; *lane 3*). Additionally, adjusted elution volume chromatograms of the thermally annealed samples (Fig. 2*D*) indicate that the various features of the refolded samples (*e.g.*, *aEV* = 0 ml) are largely absent. Instead, we see two dominant features at small-elution volumes (a*EV* ≈ - 0.31 and −0.24 ml), consistent with an increase in hydrodynamic volume resulting from the formation of ED.

Importantly, the single-molecule FRET, gel electrophoresis, and size-exclusion HPLC results from the thermally annealed samples (Fig. 2, *C* and *D*) differ substantially from those associated with the monomeric forms of 3′X55 in the refolded samples (Fig. 2, *A* and *B*). These differences indicate that there are noticeable intermolecular interactions between RNA molecules. The results of the refolded and thermally annealed samples also serve as critical reference points to characterize the spontaneous dimerization of 3′X55.

### 3′X55 spontaneously forms multiple dimeric conformations

Next, we proceeded to determine if samples containing 3′X55 could form dimers without thermal annealing. To accomplish this, we mixed ^L^3′X55 (100 pM) with refolded ^U^3′X55 (10 μM) and let the samples equilibrate for 0.5 days at room temperature (∼295 K) under baseline experimental conditions. Transfer efficiency histograms from these equilibrated samples contained at least three populations (Fig. 2*E*). Furthermore, the mean transfer efficiencies of these populations are consistent with those observed in our refolded (Fig. 2*A*) and thermally annealed (Fig. 2*C*) samples.

To characterize the oligomeric status of 3′X55 in these equilibrated (*EQ*) samples, we again used FCS analyses to determine that the average diffusion time was $\left\langle \tau_{D}\right\rangle$_*EQ*_ ≈ 0.40 ± 0.01 ms. This value falls between $\left\langle \tau_{D}\right\rangle$_*RF*_ and $\left\langle \tau_{D}\right\rangle$_*TA*_, which is qualitatively consistent with some dimerization. However, a more quantitative comparison of the transfer efficiency histograms and average diffusion times highlights an apparent inconsistency. Specifically, the fractional abundance (ϕ) of the low-transfer efficiency population in the equilibrated sample is only ∼ 0.14. Therefore, the population-weighted average diffusion time (*i.e.*, 0.14 $\left\langle \tau_{D}\right\rangle$_*TA*_ + 0.86 $\left\langle \tau_{D}\right\rangle$_*RF*_ = 0.37 ± 0.01 ms) should have been smaller than the value we observed. This apparent inconsistency suggests that the low-transfer efficiency extended dimeric conformation is not the only oligomeric conformation in this sample.

Again, when monitored *via* gel electrophoresis, these equilibrated samples travel through the gel as a mixture of the monomeric and dimeric 3′X55 bands (Fig. S1*A*; *lane 4*). These results further confirm that 3′X55 will adopt both monomeric and dimeric conformations when equilibrated.

Finally, the adjusted elution volume chromatograms associated with these equilibrated samples (Fig. 2*F*) contained features that were similar to those observed in the refolded (Fig. 2*B*) and thermally annealed (Fig. 2*D*) samples. However, new features were also apparent in the chromatogram, which we attribute to other dimeric conformations of 3′X55. The most prominent of these new features was located at *aEV* ≈ −0.1 ml, which is between the features of the refolded and thermally annealed reference samples, suggesting this medium-elution volume feature is likely associated with a hydrodynamically compact dimeric conformation (CD) of 3′X55. If these molecules had high- and/or intermediate-transfer efficiency values, then the apparent inconsistency highlighted above would be resolved.

**Figure 3:**
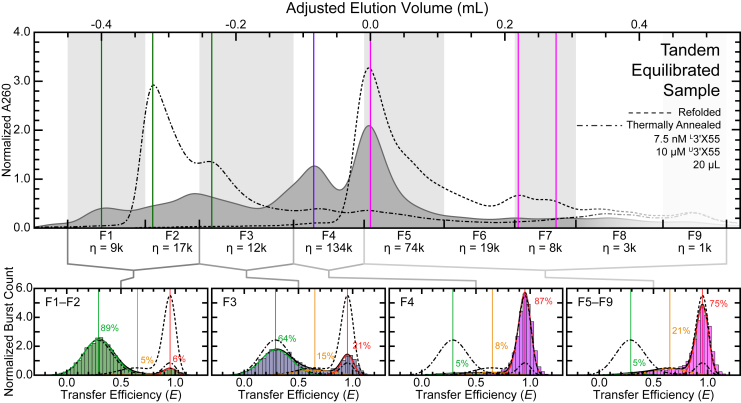
**Tandem Size-exclusion HPLC/Single-molecule FRET.** The various features of an equilibrated sample of 3′X55 can be separated and collected into fractions using size-exclusion HPLC, and then further evaluated using single-molecule FRET. The small-elution volume populations (*dark green*) collected in F1 and F2 are dominated by a low-transfer efficiency population (*light green*) and largely resemble the thermally annealed reference samples. In F3, there is a notable increase in a high-transfer efficiency population (*red*). The medium-elution volume populations (*purple*), F4, is overwhelmingly dominated by a high-transfer efficiency population of 3′X55 (*red*) and contains the highest concentration of labeled RNA based on the dilution-corrected number of bursts (η). The large-elution volume populations (*pink*) collected in F5-F9 closely resemble the refolded reference sample with a major high-transfer efficiency population (*red*) and a minor intermediate-transfer efficiency population (*orange*). Thermally annealed and refolded reference samples are shown as *broken lines*.

To further explore the possibility of dimeric species at high- and/or intermediate-transfer efficiency values, we developed an integrated approach that allowed us to conduct size-exclusion HPLC and single-molecule FRET measurements in tandem on the same sample molecules. The samples for these tandem experiments were prepared similarly to the equilibrated size-exclusion HPLC samples described above, except that they contained ^L^3′X55 and were ultimately collected in several fractions. This enabled us to first roughly separate the oligomeric populations based on their adjusted elution volumes (Fig. 3, *top*) and then acquire transfer efficiency histograms (Fig. 3, *bottom*). The adjusted elution volume chromatograms of these samples (Fig. 3, *top*) were nearly identical to those associated with the equilibrated samples (Fig. 2*F*). Furthermore, transfer efficiency histograms from the various fractions highlight several notable findings. First, transfer efficiency histograms from the last five fractions (F5-F9) are similar to those associated with refolded samples (Fig. 2*A*), indicating that the large-elution volume population of monomers adopts the 3′X55a and 3′X55b conformations. Second, transfer efficiency histograms from the first two fractions (F1-F2) are similar to those associated with thermally annealed samples (Fig. 2*C*), indicating that the small-elution volume ED population is also present. Intriguingly, histograms from fraction three (F3) show a pronounced increase in high-transfer efficiency bursts, and the histogram from fraction four (F4), which contains most of the medium-elution volume CD population of 3′X55 (Fig. 2*F*), is overwhelmingly dominated by high-transfer efficiency values. The results from F4 of the tandem experiments demonstrate that this compact dimeric conformation likely consists of two 3′X55 molecules, each likely adopting a high-transfer efficiency conformation that resembles 3′X55a. This finding also supports the proposed resolution to the apparent inconsistency described above.

### Dimerization is concentration dependent

**Figure 4:**
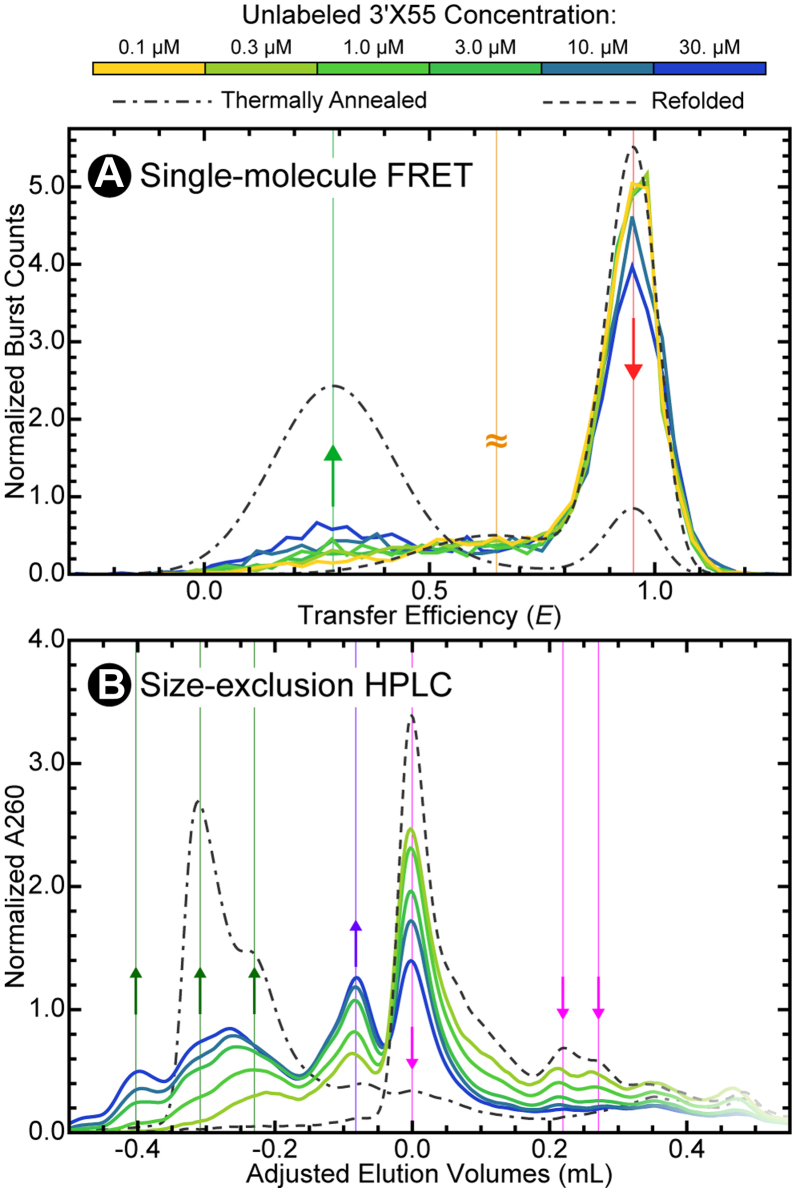
**Concentration-dependent Dimerization of 3′X55.***A*, 3′X55 will spontaneously dimerize with higher concentrations of RNA, resulting in a greater abundance of the low-transfer efficiency population (*light green*). *B*, the small- and medium-elution volume populations (*dark green* and *purple*) all increase with increasing concentrations of 3′X55. Conversely, the large-elution volume populations (*pink*) decrease as the total RNA concentration increases. Thermally annealed and refolded reference histograms and chromatograms are shown as *broken lines*.

Knowing that 3′X55 can dimerize when equilibrated at room temperature, we set out to monitor this process over a range of RNA concentrations under baseline experimental conditions. Transfer efficiency histograms (Fig. 4*A*) from our single-molecule FRET experiments demonstrate that the low-transfer efficiency population associated with the ED becomes increasingly more abundant as the ^U^3′X55 concentration increases to 30 μM. However, our ability to monitor ^L^3′X55 dimerization *via* FRET is somewhat limited because the high-transfer efficiency population is made up of both monomeric and dimeric species. Fortunately, size-exclusion HPLC is better suited for this task due to its ability to more clearly determine the abundance of the various oligomeric species. As expected, the adjusted elution volume chromatograms at high concentrations of ^U^3′X55 (Fig. 4*B*) indeed depict an increased abundance of RNAs at small-elution volumes, particularly those associated with the ED populations of the thermally annealed sample. Additionally, they also show an increase in the abundance of the medium-elution volume population associated with the CD as a function of RNA concentration, which further supports the notion that this is indeed a dimeric conformation of 3′X55 that also happens to be more hydrodynamically compact than the ED (Fig. 4*B*).

### Formation of the compact dimer is magnesium-dependent

**Figure 5:**
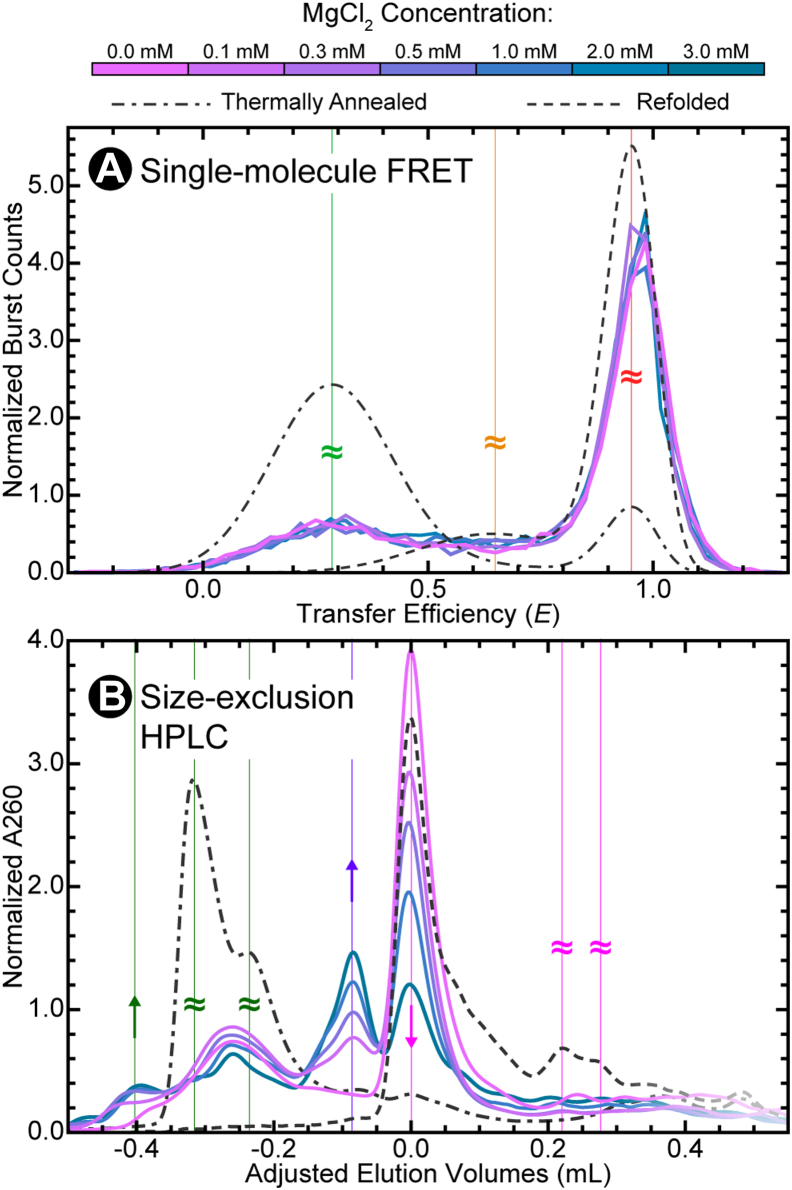
**MgCl_2_-dependent 3′X55 Dimerization.***A*, overlay of transfer efficiency histograms where ^L^3′X55 was equilibrated in the presence of 10 μM ^U^3′X55 at various physiologically inspired concentrations of MgCl_2_. The low-transfer efficiency ED population (*light green*) is equally abundant in all samples. *B*, overlay of adjusted elution volume chromatograms where 10 μM ^U^3′X55 was equilibrated at various concentrations of MgCl_2_. The small-elution volume ED populations (*dark green*) are present in all samples regardless of MgCl_2_ concentration. Conversely, the abundance of the medium-elution volume CD population (*purple*) is largely absent in the absence of MgCl_2_ and increases systematically as the concentration of MgCl_2_ increases. Refolded and thermally annealed reference samples performed at 1 mM MgCl_2_ are shown as *broken lines*.

Our previous single-molecule FRET work demonstrated that the intrinsic conformational equilibrium of monomeric 3′X55 is influenced by MgCl_2_ concentration (18). Here, we aimed to investigate the effect of MgCl_2_ concentration on dimerization. Curiously, the transfer efficiency histograms resulting from our MgCl_2_ titrations are remarkably similar (Fig. 5*A*), suggesting that the formation of the low-transfer efficiency ED is not strongly influenced by these divalent counterions. This finding was also supported by size-exclusion HPLC (Fig. 5*B*), where the abundance of the small-elution volume ED is largely independent of the MgCl_2_ concentration. Interestingly, these adjusted elution volume chromatograms also showed how the abundance of the medium-elution volume CD greatly increases at elevated concentrations of MgCl_2_ (Fig. 5*B*). Together, these results demonstrate that the pathways that give rise to ED and CD have different MgCl_2_ requirements.

### Dimerization is slow

**Figure 6:**
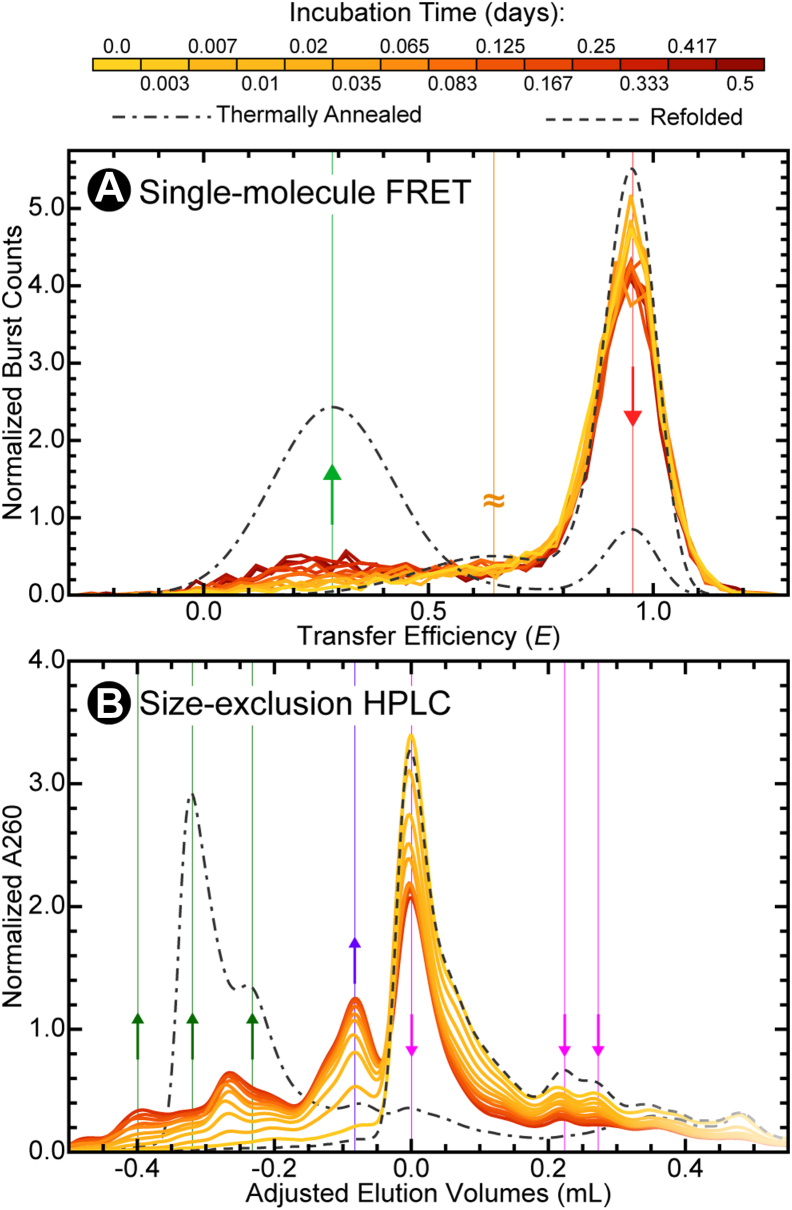
**Time-dependent Dimerization of 3′X55.***A*, overlay of transfer efficiency histograms from samples containing 10 μM ^U^3′X55 at various time points over the course of 0.5 days (*yellow* to *maroon*). The low-transfer efficiency population (*light green*) increases over time, whereas the high-transfer efficiency population (*red*) decreases over time. *B*, overlay of adjusted elution volume chromatograms from samples containing 10 μM ^U^3′X55 at various time points over the course of 0.25 days. The abundances of the small- and medium-elution volume dimeric populations (*dark green and purple*) increase over time, whereas the abundances of the large-elution volume monomeric populations (*pink*) decrease over time. Thermally annealed and refolded reference samples are shown as *broken lines*.

Finally, we wanted to gain kinetic insights into the mechanisms governing dimerization. Therefore, we monitored the consumption and production of the various monomeric and dimeric species as a function of time using both single-molecule FRET and size-exclusion HPLC under baseline experimental conditions. The transfer efficiency histograms (Fig. 6*A*) showed a slight but systematic increase in the low-transfer efficiency population associated with the extended dimeric conformation of 3′X55 over the course of 0.5 days. Similarly, the adjusted elution volume chromatograms also change over a comparable period of time (Fig. 6*B*), highlighting the appearance of both the ED and CD populations of 3′X55. These results show that 3′X55 monomers are slowly consumed over several hours to form both dimeric species.

## Discussion

In this study, we showed that 3′X55 adopts multiple dimeric conformations at high RNA concentrations. Single-molecule FRET and size-exclusion HPLC were used to characterize the structural, energetic, and dynamic properties of both the monomeric and dimeric forms of this RNA. Consistent with previous results (18), our refolded samples (Fig. 2, *A* and *B*) demonstrated that at low concentrations, 3′X55 adopts two distinct monomeric conformations (*i.e.*, 3′X55a and 3′X55b). Our thermally annealed samples (Fig. 2, *C* and *D*) indicated that at higher concentrations, 3′X55 can form a low-transfer efficiency extended dimeric conformation (ED) that is likely stabilized by many intermolecular base-pairing interactions (Fig. 1*D*, *right*) (22, 23, 29). Additionally, our equilibrated samples (Figs. 2, *E* and *F*, 3, and 4) revealed that 3′X55 spontaneously dimerizes at high RNA concentrations, forming this low-transfer efficiency ED as well as a high-transfer efficiency compact dimeric conformation (CD) when MgCl_2_ is present (Fig. 5). However, when MgCl_2_ is absent, we only observed ED, which suggests that the observed CD is not an essential intermediate for the formation of ED and, consequently, that the formation of ED likely precedes the formation of CD.

**Figure 7:**
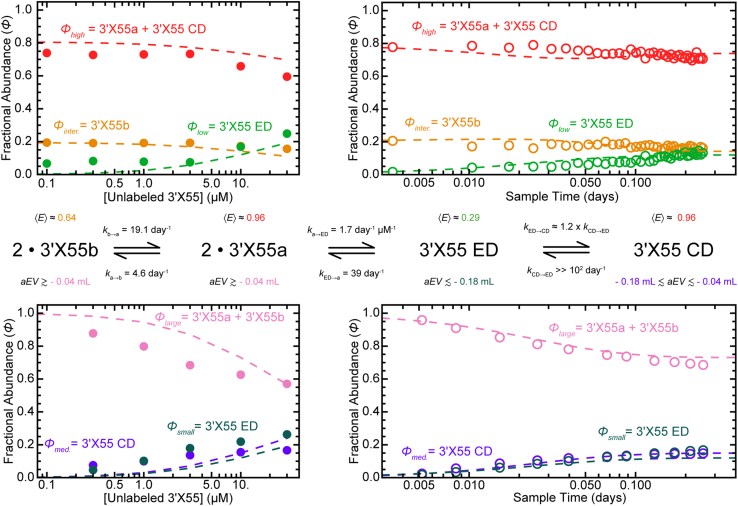
**A simple four-state kinetic model for the dimerization of 3′X55.** Numerical analysis methods were used to approximate (*brokenlines*) the concentration (*left*) and time (*right*) dependence of dimerization observed in our single-molecule FRET (*top*) and size-exclusion HPLC (*bottom*) experiments. Optimal values for the rate constants (*middle*) were found by systematically adjusting the kinetic parameters to maximize concurrence between the model and our experimental data. Due to the limited ability to separate all the conformations in single-molecule FRET measurements, we assigned 3′X55a and the compact dimeric conformation (CD) to the high-transfer efficiency population (*red*), 3′X55b was assigned to the intermediate-transfer efficiency population (*orange*), and the extended dimeric conformation (ED) was assigned to the low-transfer efficiency population (*green*). We also assigned 3′X55a and 3′X55b as the large-elution volume populations (pink), CD was assigned to the medium-elution volume population (*purple*), and the ED was assigned to the small-elution volume population (*dark green*).

To better understand the intricate coupling of these monomeric and dimeric conformational equilibria, we conducted several concentration- and time-dependent experiments under baseline experimental conditions. Like any oligomerization process, the dimerization of 3′X55 was highly concentration-dependent (Fig. 4). From the concentration-dependent datasets, we quantified the fractional abundance (ϕ) of the low-, intermediate-, and high-transfer efficiency populations as well as the small-, medium-, and large-elution volume populations at equilibrium as a function of 3′X55 concentration (Figs. 4 and 7, *left*). The same approach was used to quantify the fractional abundances of the same populations in our time-dependent datasets (Figs. 6 and 7, *right*). Then, we developed a simple four-state kinetic model that we could numerically analyze to determine the concentration- and time-dependence of dimerization (Fig. 7, *middle*). Two of the kinetic rate constants in this model (*i.e.*, $k_{a\to b}$ and $k_{b\to a}$) were independently determined under these conditions (Fig. S2). The other four kinetic rate constants (*i.e.*, $k_{a\to ED}$, $k_{ED\to a}$, $k_{ED\to CD}$, and $k_{CD\to ED}$) were systematically adjusted during the numerical analysis of our kinetic model to maximize the model’s concurrence with both the concentration- and time-dependent datasets from both the single-molecule FRET and size-exclusion HPLC experiments (Fig. 7).

The concurrence between our experimental results and the optimized kinetic model highlights several notable aspects of the 3′X55 dimerization mechanism under baseline experimental conditions. (*i*) The conformation of monomeric 3′X55 that presents its dimer linkage sequence as an apical loop (3′X55a) can interact with itself to form dimers. (*ii*) The equilibrium dissociation constant for dimerization is ∼ 23 μM; this likely helps to ensure that most viral genomes remain monomeric, and, thus, primed for either protein synthesis or RNA replication (25, 26, 27), until the later stages of infection. Curiously, previous studies of two 3′X55 mutants optimized for heterodimerization reported sub-micromolar binding affinities, highlighting the pronounced impact of subtle sequence modifications in this highly conserved RNA (28). (*iii*) The rate constant for the dissociation of the dimeric species is quite small, which ensures that 3′X remains dimeric for an extended period of time once these intermolecular interactions have been formed. (*iv*) The two dimeric conformations resulting from this homomeric interaction (*i.e.*, ED and CD) have nearly identical thermodynamic stabilities under baseline experimental conditions (*K*_*eq*_ ≈ 1), which is curious given their pronounced structural differences. While the CD we observe may appear structurally consistent with a previously reported kissing complex (28) (Fig. 1*D*, *left*), the lifetime of such a transient intermediate would likely be orders of magnitude shorter than what our kinetic model suggests. While a kissing complex may certainly exist, we feel it would likely be too short-lived to detect in our experiments. Alternatively, we feel that the slow breakdown of the initial dimeric state is more compatible with ED, which would be held together by substantially more intermolecular interactions. Additionally, we propose that the MgCl_2_-dependent CD we observe is distinct from any previously proposed kissing complexes and speculate that it may resemble a cruciform or zipper structure (36) where the DLS remains in an intermolecular stem-loop structure (Fig. 1*D*, *middle*). Curiously, this would leave the dimer linkage sequences of the CD free to interact with other copies of the viral genome, thus enabling the formation of higher-order oligomeric states. Trace amounts of these oligomeric species may even be present in our adjusted elution volume chromatograms at the smallest elution volumes (Figs. 2*F*, 3, 4*B*, 5*B*, and 6*B*).

While other research groups have speculated about the biological significance of genome dimerization (28, 37), we feel that the aspects of dimerization highlighted above are consistent with a novel functional role in aiding the transition from biomolecular synthesis to viral assembly. Specifically, we propose that dimerization could act as a genome concentration-dependent mechanism to deplete the two monomeric forms of 3′X, which themselves have been implicated in either viral protein synthesis or (−) strand RNA synthesis (25, 26, 27). Therefore, genome dimerization would function as a queue to shut down these biosynthetic processes by preventing ribosomes and polymerases from loading onto the genomes during the later stages of infection. Consequently, the viral genomes would become more readily available to associate with nucleocapsid proteins to eventually form viral particles. While the small dissociation rate constant we observe would likely keep the viral genomes in a dimeric state for an extended period, the small association rate constant may be too slow to shut down these biosynthetic processes on a relevant timescale. However, the HCV nucleocapsid protein, which is also present at high concentrations during the later stages of infection, has been shown to significantly enhance dimerization of 3′X (22, 28, 38), which is likely to greatly accelerate the transition from biomolecular synthesis to viral assembly.

In conclusion, we utilized an integrative combination of single-molecule FRET and size-exclusion HPLC to demonstrate that 3′X55 spontaneously dimerizes at high RNA concentrations, adopting both compact and extended dimeric conformations. We have quantitatively characterized how this process depends on the concentrations of both RNA and MgCl_2_. Additionally, we saw that dimerization is exceptionally slow, taking several hours to reach equilibrium. Our proposed mechanism for this process is described by a simple four-state kinetic model that is remarkably consistent with the experimental data. Overall, these mechanistic insights further support the emerging hypothesis that this highly conserved, untranslated RNA is a multifaceted riboregulatory element and that its inherent ability to dimerize could allow it to shut down other RNA-dependent processes during the later stages of infection.

## Experimental procedures

### Chemicals and reagents

Ultrapure water (18.2 MΩ cm) was used to prepare all aqueous solutions. Sodium mono- and dibasic phosphate, 2-[4-(2-hydroxyethyl)piperazin-1-yl]ethanesulfonic acid (HEPES), Ethylenediaminetetraacetic acid (EDTA), Tween 20, urea, dimethyl sulfoxide, and magnesium chloride were all sourced from Sigma-Aldrich. Sodium hydroxide, sodium chloride, and acetonitrile (ACN) were all purchased from Fisher Scientific. Triethylammonium acetate (TEAA) was purchased from Millipore. N-hydroxysuccinimide ester-modified fluorescent dyes Cy3B (NHS-Cy3B) and CF660R (NHS-CF660R) were purchased from Cytiva and Sigma-Aldrich, respectively. T4 RNA Ligase II and DNase I enzymes were purchased from New England Biolabs. All RNA and DNA oligonucleotides were custom ordered from Integrated DNA Technologies (Table S1).

### Preparation of fluorescently labeled and unlabeled 3′X55 RNA

The design, synthesis, and purification of the fluorescently labeled 3′X55 construct used in this study (^L^3′X55) have been described previously (18). Briefly, ^L^3′X55 was synthesized *via* the splinted ligation (39) of two synthetic RNA oligonucleotides (Table S1). RNA one consists of the first 29 nucleotides of 3′X55 (1–29) with an internal amino-modified dT replacing U13 (Table S1). This oligo was labeled with N-hydroxysuccinimide ester (NHS-ester) modified Cy3B fluorescent dye. RNA two consists of the remaining 26 nucleotides of 3′X55 (30–55) along with a 5′ phosphate and an internal amino-modified phosphate located between nucleotides 39 and 40 (Table S1), which was labeled with an NHS-ester modified CF660R fluorescent dye. Once labeled, the two RNA oligos were purified using reverse-phase HPLC, annealed to a fully complementary DNA splint (Table S1), ligated with T4 RNA Ligase II, and then treated with DNase I to digest the splint. The resulting ^L^3′X55 was again purified with HPLC and then resuspended in storage solution (10 mM HEPES, 5 mM NaOH, and 0.1 mM EDTA) at a final RNA concentration of either 100 nM or 10 nM. The 10 nM stocks of ^L^3′X55 were then refolded by incubating at 95 °C for 20 min prior to plunging the stock solution in liquid nitrogen for 10 min to limit the formation of higher-order structures. The unlabeled variant of 3′X55 (^U^3′X55) was ordered as a single RNA oligo (Table S1). Before use, this analytically verified oligo (Fig. S3) was dissolved in storage solution at a final RNA concentration of either 178 μM or 196 μM. All stocks of ^L^3′X55 and ^U^3′X55 were stored at −80 °C.

### Single-molecule FRET samples and data acquisition/analysis

Refolded single-molecule FRET samples were prepared to achieve baseline experimental conditions (*i.e.*, 1 mM MgCl_2_, 150 mM NaCl, 25 mM HEPES, 12.5 mM NaOH) using an aliquot of ^L^3′X55 refolded at 10 nM (see above). Thermally annealed single-molecule FRET samples were prepared using a no-magnesium solution (150 mM NaCl, 25 mM HEPES, 12.5 mM NaOH) containing 20 μM ^U^3′X55 and 200 pM ^L^3′X55. Samples were then incubated at 95 °C for 20 min and then slowly cooled to 35 °C over the course of 2 h on the heating block, at which point they were further diluted with solutions to achieve baseline experimental conditions. Equilibrated single-molecule FRET samples were prepared by first refolding solutions of ^U^3′X55 at twice the desired final concentration (0.2 μM–60 μM). The volume was then doubled using an aliquot of ^L^3′X55 refolded at 10 nM (see above) and various stock solutions to achieve the desired solution conditions. The resulting samples were then evaluated after equilibrating at ∼ 295 K for more than 0.5 days. The detergent Tween 20 was included in all single-molecule FRET samples at a concentration of 50 μM to passivate the surfaces of the sample holders and minimize surface adsorption of 3′X55.

Most aspects of the single-molecule FRET data acquisition and analysis have been described previously (18). One notable difference is that instead of using alternating laser excitation (40), we used pulsed interleaved excitation (41), where each monochromatic laser (515 nm and 642 nm) emits a single ∼ 100 ps pulse of light every ∼ 50 ns, with the two excitation sources temporally offset by ∼ 25 ns (41). The data were collected in 10-min fluorescence time trajectories, which were analyzed using methods from fluorescence correlation spectroscopy (FCS) to study the dynamics of sub-millisecond processes (42). Specifically, FCS was used to determine the mean diffusion time, ⟨*τ*_*D*_⟩, of ^L^3′X55 by fitting the fluorescence autocorrelation function, *G*(*τ*), resulting from 642 nm excitation to an equation describing molecular diffusion (40) (Equation 1).

(1) G(τ)=G01(1+(10τ10⟨τD⟩)(1+7−2(10τ10⟨τD⟩))12+1

In addition to the FCS analyses, we also conducted burst analyses of fluorescence time trajectories. Here, individual photon arrival times were partitioned into 1 ms time bins. Time bins with > 30 photons were classified as bursts of fluorescence and used to calculate fluorophore stoichiometry (*S*) and transfer efficiency (*E*) values. Any bursts with an *S* value that was between 0.25 and 0.75 were considered to have arisen from FRET-labeled molecules (40, 41). These bursts were further refined to select only those with > 40 photons after all correction factors had been applied. These so-called high-quality bursts were used to generate transfer efficiency histograms, which were fit to a sum of three distributions—two Gaussian distributions (low-transfer efficiency and intermediate-transfer efficiency) and a log-normal distribution (high-transfer efficiency)—to determine the mean transfer efficiency, ⟨*E*⟩, and fractional abundance (*ϕ*) of each distribution.

### Polyacrylamide gel electrophoresis (PAGE) samples and data acquisition/analysis

Refolded PAGE samples were prepared by first diluting ^U^3′X55 to twice the desired final concentration (1 μM and 20 μM) with water and then incubating at 95 °C for 20 min prior to plunging the sample in liquid nitrogen for 10 min to limit the formation of higher-order structures. Upon thawing, the volume was doubled to achieve baseline experimental conditions and then loaded onto the gel. Thermally annealed PAGE samples were prepared using a no-magnesium solution containing 20 μM ^U^3′X55. Samples were then incubated at 95 °C for 20 min and then slowly cooled to 35 °C over the course of 2 hours on the heating block, at which point they were further diluted with solutions to achieve baseline experimental conditions. Equilibrated PAGE samples were prepared by first refolding samples of ^U^3′X55 at twice the desired final concentration (20 μM). The volume was then doubled to achieve baseline experimental conditions and allowed to equilibrate at ∼ 295 K for more than 0.5 days before being loaded onto the gel.

Bis-Tris 4 to 12% polyacrylamide gels (Invitrogen) were first run without any samples at 100V for 30 min under baseline experimental conditions to wash out the storage buffer. Then, the refolded, thermally annealed, and equilibrated samples were loaded into the wells of the equilibrated gels using 30% v/v glycerol. Gels were run under baseline experimental conditions at 100V for 1 h with the chamber placed in an ice-water bath. The gels were then stained in a 3× solution of GelRed (Biotium) for an hour and imaged using an Odyssey M (LICOR). The resulting images were imported into Mathematica (Wolfram Alpha) for analysis. The mean pixel intensity down each lane was determined by integrating across the width of each lane. Next, the background was determined by taking the average of the mean pixel intensity values associated with the first 10 and last 15 pixels down each lane. Finally, the background-corrected data were normalized based on the integrated mean pixel intensity of each lane.

### Size-exclusion HPLC samples and data acquisition/analysis

Refolded size-exclusion HPLC samples were prepared by first diluting ^U^3′X55 to twice the desired final concentration (1 μM) with water and then incubating at 95 °C for 20 min prior to plunging the sample in liquid nitrogen for 10 min to limit the formation of higher-order structures. Upon thawing, the volume was doubled to achieve baseline experimental conditions and then immediately evaluated. Thermally annealed size-exclusion HPLC samples were prepared using a no-magnesium solution containing 20 μM ^U^3′X55. Samples were then incubated at 95 °C for 20 min and then slowly cooled to 35 °C over the course of 2 h on the heating block, at which point they were further diluted to achieve baseline experimental conditions. Equilibrated size-exclusion HPLC samples were prepared by first refolding samples of ^U^3′X55 at twice the desired final concentration (0.6 μM – 60 μM). The volume was then doubled using a solution to achieve the desired experimental conditions. The resulting samples were then evaluated after equilibrating at ∼ 295 K for at least 0.5 days.

Size-exclusion HPLC data acquisition was performed at a flow rate of 0.35 ml/min using a TSKgel UP0SW2000 column (Tosoh Biosciences) attached to the same HPLC system that was previously used to prepare the fluorescently labeled RNA (18). In all experiments, the composition of the running buffer matched that of the sample. Raw chromatograms were generated by monitoring the absorbance of each filtered (Millex-GV 0.22 μm PVDF, Millipore) sample at 260 nm for 20 min after injection. The temporal position of the local maxima associated with the monomeric population of a given raw chromatogram was identified and then used to generate adjusted elution time chromatograms by subtracting the value of the maxima from all other time points. To facilitate comparisons across different MgCl_2_ concentrations (Fig. 5), the time points were further adjusted using a scaler to account for differences in elution times resulting from subtle changes in electrostatic interactions between the RNA and the size-exclusion column. Lastly, the adjusted elution times were multiplied by the flow rate to generate adjusted elution volume (*aEV*) chromatograms, which were then normalized by the integrated absorbance to account for slightly different amounts of material in each injection.

To roughly quantify the fractional abundance (*ϕ*) of the various populations present in an adjusted elution volume chromatogram, we calculated integrated absorbance values across three different elution bands: the small-elution volume population below - 0.18 ml was attributed to molecules in the extended dimeric conformation (ED), the medium-elution volume population between −0.18 ml and −0.04 ml was attributed to molecules in the compact dimeric conformation (CD), and the large-elution volume population above −0.04 ml was attributed to monomeric molecules in either the 3′X55a or 3′X55b conformation. These boundaries correspond to the local *aEV* minima that separate the various populations in the chromatogram resulting from the equilibrated sample under baseline experimental conditions (Fig. 2*F*).

### Tandem Size-exclusion HPLC/single-molecule FRET samples

The tandem samples were prepared using a solution containing 20 μM ^U^3′X55 and 15 nM ^L^3′X55. Samples were incubated at 95 °C for 20 min prior to plunging the solution in liquid nitrogen for 10 min to limit the formation of higher-order structures. Next, the samples were thawed and further diluted to achieve baseline experimental conditions and then injected after equilibrating at ∼ 295 K for at least 0.5 days. The eluant was collected in ∼ 100 μl fractions, each containing near single-molecule concentrations ^L^3′X55. Fractions were then supplemented with Tween 20 to achieve a final concentration of 50 μM prior to being plunged in liquid nitrogen to preserve the samples until the single-molecule FRET distribution of the fraction could be measured.

### Kinetic measurements

The single-molecule FRET samples used to monitor the kinetics of dimerization were prepared by first refolding a 20 μM solution of ^U^3′X55 before doubling the volume with an aliquot of ^L^3′X55 refolded at 10 nM (see above) and various stock solutions to achieve baseline experimental conditions. The acquisition of single-molecule fluorescence time trajectories began immediately after the addition of ^L^3′X55 and continued in 10-min increments for 0.5 days. Size-exclusion HPLC kinetics samples were prepared similarly, except that they lacked ^L^3′X55. Here, measurements at specific time-points were achieved by removing 1 μl aliquots from the reaction mixture after the desired amount of time. These aliquots were then mixed with 19 μl of size-exclusion HPLC running buffer and frozen in liquid nitrogen until they could be thawed and evaluated on the HPLC.

## Numerical analysis of dimerization

The time- and RNA concentration-dependence of dimerization observed in the single-molecule FRET and size-exclusion HPLC experiments were globally modeled *via* the numerical approximation of a simple four-state kinetic model described by the following system of equations and appropriate initial conditions.

(2) $$\frac{d}{dt}\left[\begin{array}{c} \left[3^{\prime }X55b\right]\\ \left[3^{\prime }X55a\right]\\ \left[ED\right]\\ \left[CD\right] \end{array}\right]=\left[\begin{array}{cccc} -k_{b\to a} & k_{a\to b} & 0 & 0\\ k_{b\to a} & -k_{a\to b}-2\left[3^{\prime }X55a\left(t\right)\right]k_{a\to ED} & 2k_{ED\to a} & 0\\ 0 & \left[3^{\prime }X55a\left(t\right)\right]k_{a\to ED} & -k_{ED\to a}-k_{ED\to CD} & k_{CD\to ED}\\ 0 & 0 & k_{ED\to CD} & {-k}_{CD\to ED} \end{array}\right]\cdot \left[\begin{array}{c} \left[3^{\prime }X55b\left(t\right)\right]\\ \left[3^{\prime }X55a\left(t\right)\right]\\ \left[ED\left(t\right)\right]\\ {}[CD\left(t\right) \end{array}\right]$$

Here, the rate of concentration change, $\frac{d\;}{dt}\left[x\right]$, in conjunction with the time step, dt, determines the concentration, [x(t)], of the next logarithmically spaced time point. The resulting concentrations were used to determine the fractional abundance (ϕ) of each species in a manner that was consistent with either the single-molecule FRET or the size-exclusion HPLC measurements.

## Data availability

Data supporting the findings of this manuscript are available from the corresponding authors upon reasonable request.

## Supporting information

This article contains supporting information (18).

## Conflict of interest

The authors declare that they do not have any conflicts of interest with the content of this article.

## Supporting information

Supporting infromation
