# Supplementary material for: Spontaneous dimerization of the hepatitis C virus 3′X RNA
Source: J Biol Chem. 2025 Nov 20;302(1):110963. doi: 10.1016/j.jbc.2025.110963 (PMC12809093; doi:10.1016/j.jbc.2025.110963)
Supplement: Supporting infromation [file mmc1.docx]

**SUPPORTING INFORMATION FOR:**

**Spontaneous Dimerization of the Hepatitis C Virus 3′X RNA**

Parker D. Sperstad^1^ & Erik D. Holmstrom*^1,2^

^1^ - University of Kansas, Department of Molecular Biosciences, Lawrence, KS 66045, USA

^2^ - University of Kansas, Department of Chemistry, Lawrence, KS 66045, USA

* - For correspondence: Erik Holmstrom, edh@ku.edu.

| **Oligonucleotide** | **Sequence** |
| --- | --- |
| ^U^3′X55 | 5′— GGUGG CUCCA UCUUA GCCCU AGUCA CGGCU AGCUG UGAAA GGUCC GUGAG CCGCU —3′ |
| RNA 1 | 5′— GGUGG CUCCA UCtUA GCCCU AGUCA CGGC —3′ |
| RNA 2 | 5′— pUAGCU GUGAA LAGGU CCGUG AGCCG CU —3′ |
| DNA splint | 5′— AGCGG CTCAC GGACC TTTCA CAGCT AGCCG TGACT AGGGC TAAGA TGGAG CCACC —3′ |

**Supplemental Table 1: Oligonucleotides used in this Study.** All oligonucleotides used in this study were custom ordered from IDT. The reference RNA sequence was derived from the viral genome associated with GenBank accession number AJ238799.1. The unlabeled 3′X55 (^U^3′X55) oligonucleotide is comprised of the first 55 nucleotides of 3′X. The RNA 1 (i.e., 3′X1‑29) and RNA 2 (i.e., 3′X30‑55) oligonucleotides were covalently coupled to fluorophores via primary amine modifications (i.e., t = /iAm6CT/ and L = /iUniAmM/) that were introduced during chemical synthesis. Then, RNA 1 and RNA 2 were ligated together using T4 RNA ligase II and the DNA splint to form fluorescently labeled 3′X55 (^L^3′X55).


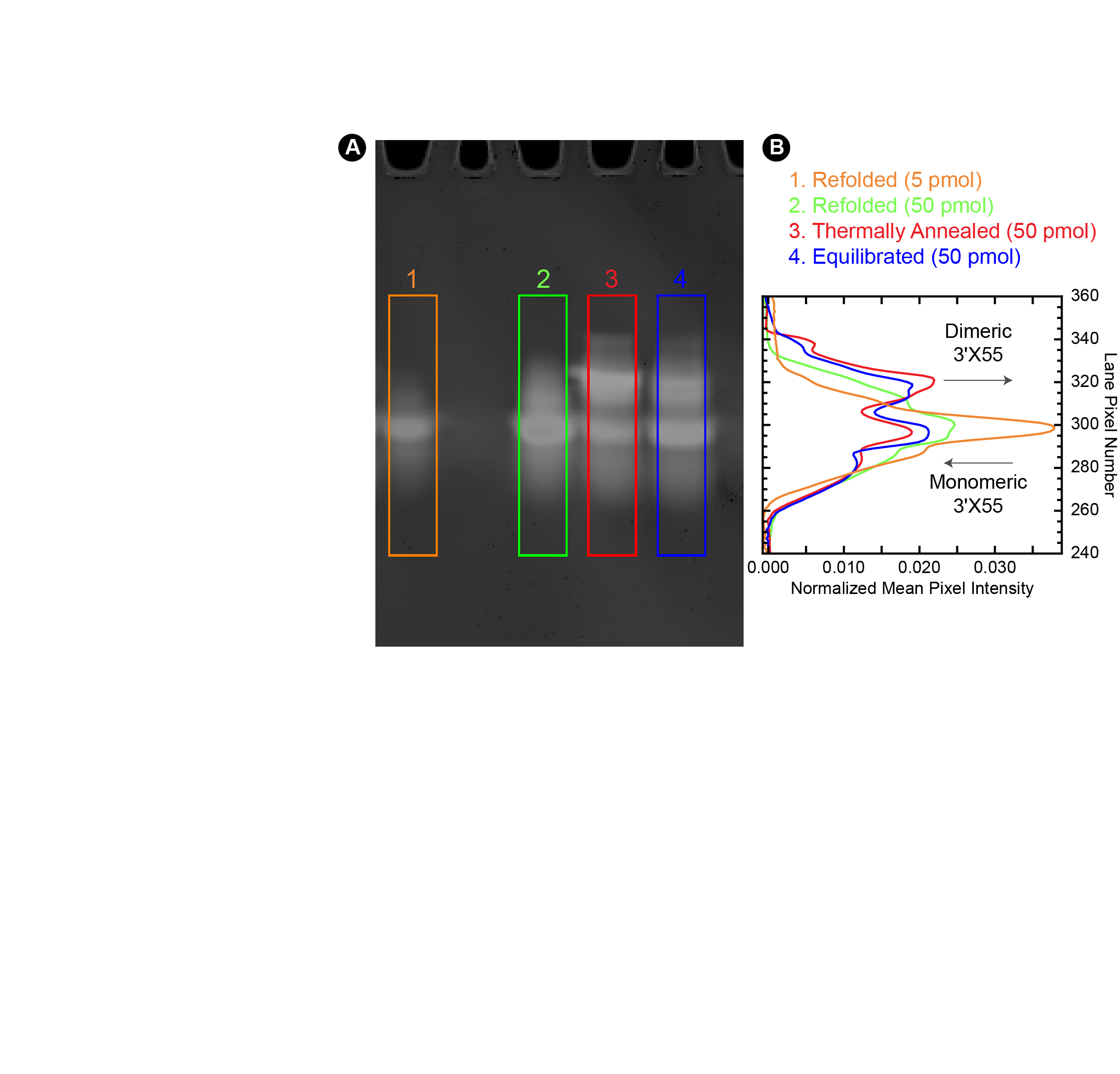


**Supplemental Figure 1: Polyacrylamide Gel Electrophoresis Analysis of ^U^3′X55 Dimerization.** (A) Refolded (*lane 1*: 5 pmol of 500 nM, *lane 2*: 50 pmol of 10 µM), thermally annealed (*lane 3*: 50 pmol of 10 µM), and equilibrated (*lane 4*: 50 pmol of 10 µM) samples in a 4-12% polyacrylamide gel. The two refolded samples appear to travel through the gel as monomeric bands. The thermally annealed sample primarily travels as dimeric bands. The equilibrated sample runs as a mixture of both monomeric and dimeric bands. The last three lanes of the gel have similar amounts of RNA in each lane, resulting in the same signal intensity when imaging. (B) The normalized mean pixel intensity of each lane reveals that the thermally annealed sample (*lane 3*, *red*) has the most dimeric RNA, whereas the more dilute refolded sample (*lane 1*, *orange*) has the most monomeric RNA. The equilibrated sample (*lane 4*, *blue*) has similar amounts of monomeric and dimeric 3′X55. The normalized mean pixel intensity also makes the higher order oligomers more obvious, which travel even slower through the polyacrylamide gel. However, several challenges are associated with quantitatively monitoring dimerization via gel electrophoresis: (1) Resistive heating makes it challenging to control the temperature; (2) The unequal staining of single-stranded and double-stranded regions of the RNA makes it difficult to quantify the relative abundance of each population; (3) The resolution of the bands is poor in running buffers that mimic our baseline experimental conditions; and (4) The amount of time it takes to run the gel greatly limits our temporal resolution. Therefore, we limit our quantitative analyses to the more robust dataset resulting from single-molecule FRET and size-exclusion HPLC.

**
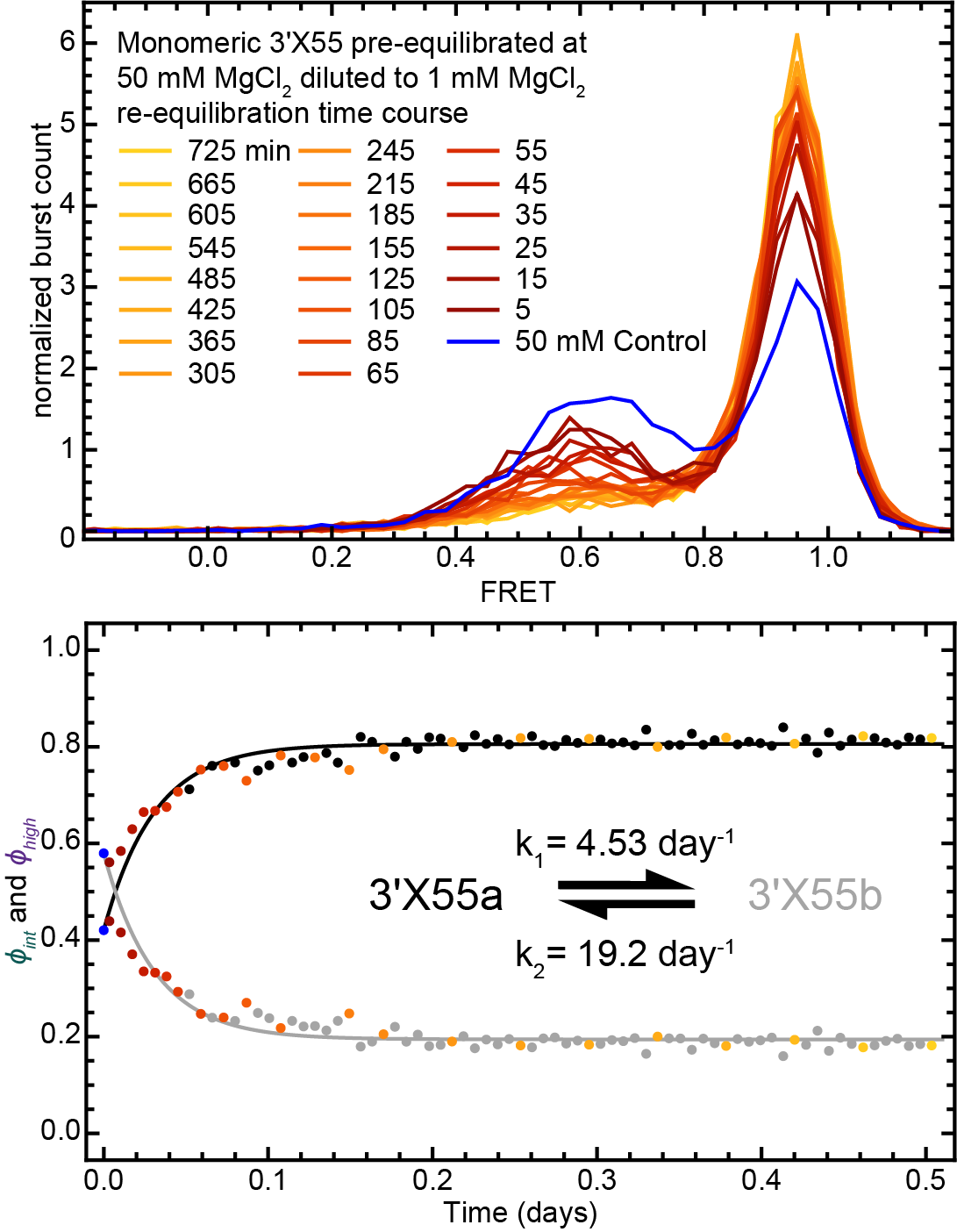
**

**Supplemental Figure 2: Determining Rate Constants for monomeric interconversion at 1 mM MgCl_2_.** To determine the monomeric interconversion rates, a sample was pre-equilibrated at 50 mM MgCl_2_ and then diluted 1:50 with 150 mM NaCl, 25 mM HEPES, 12.5 mM NaOH, and 50 µM TWEEN® 20. The sample was measured for 0.5 days using alternating laser excitation. The fractional abundance of the high- and intermediate-transfer efficiency populations was plotted as a function of time and fitted as previously described ^18^. The colors of select transfer efficiency histograms (top) match the colors of the corresponding data points in the fractional abundance plot (bottom).


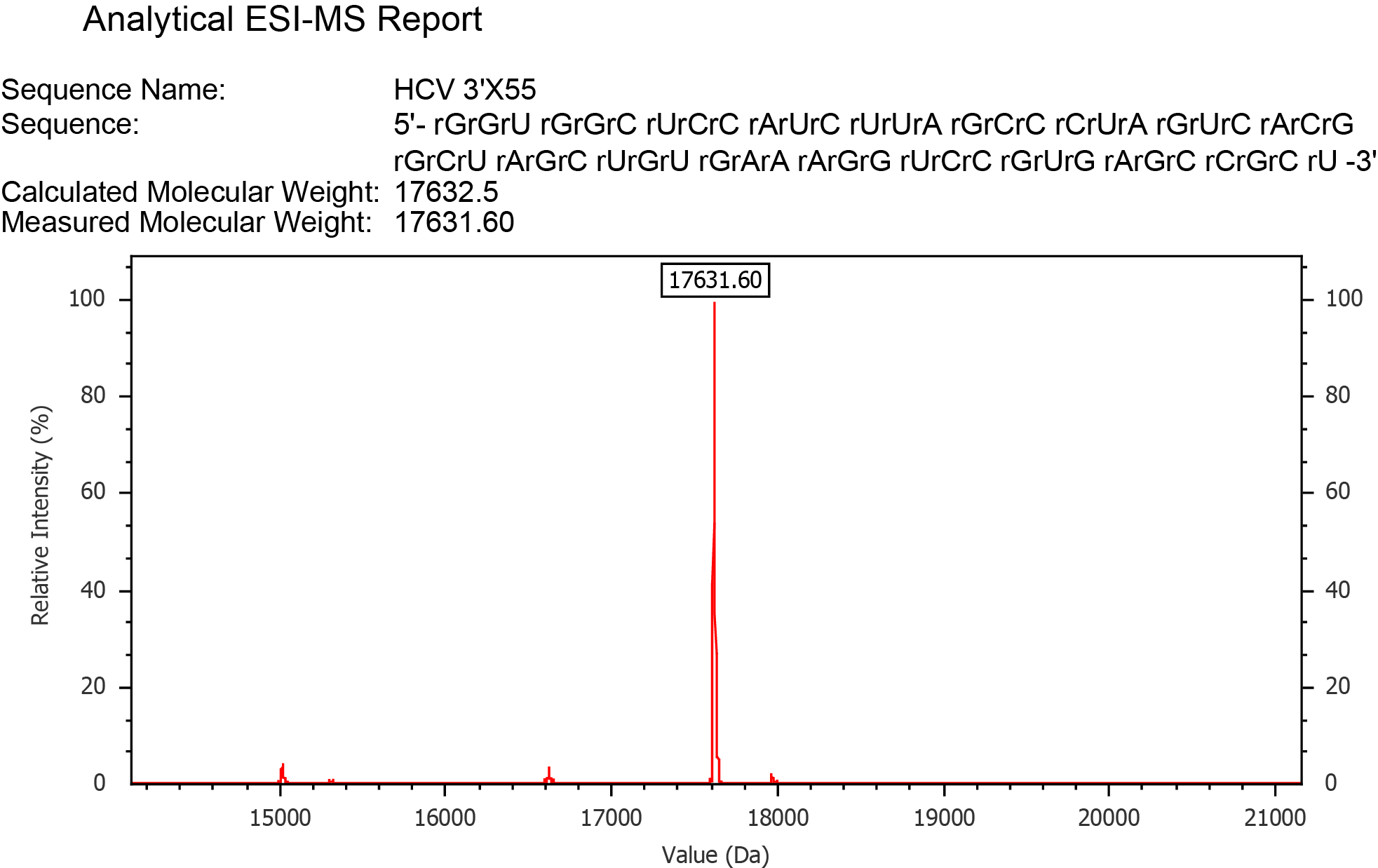


**Supplemental Figure 3: Mass spectrum of ^U^3′X55.** ESI-MS QC report shows that chemically synthesized ^U^3′X55 was within a single Dalton (Da) of the expected molecular weight. Furthermore, the readout shows that the final synthesis product is largely free of impurities.
